# Differential Immunotoxicity Induced by Two Different Windows of Developmental Trichloroethylene Exposure

**DOI:** 10.1155/2014/982073

**Published:** 2014-02-20

**Authors:** Kathleen M. Gilbert, William Woodruff, Sarah J. Blossom

**Affiliations:** University of Arkansas for Medical Sciences, Arkansas Children's Hospital Research Institute, 13 Children's Way, Little Rock, AR 72202, USA

## Abstract

Developmental exposure to environmental toxicants may induce immune system alterations that contribute to adult stage autoimmune disease. We have shown that continuous exposure of MRL+/+ mice to trichloroethylene (TCE) from gestational day (GD) 0 to postnatal day (PND) 49 alters several aspects of CD4^+^ T cell function. This window of exposure corresponds to conception-adolescence/young adulthood in humans. More narrowly defining the window of TCE developmental exposure causes immunotoxicity that would establish the stage at which avoidance and/or intervention would be most effective. The current study divided continuous TCE exposure into two separate windows, namely, gestation only (GD0 to birth (PND0)) and early-life only (PND0-PND49). The mice were examined for specific alterations in CD4^+^ T cell function at PND49. One potentially long-lasting effect of developmental exposure, alterations in retrotransposon expression indicative of epigenetic alterations, was found in peripheral CD4^+^ T cells from both sets of developmentally exposed mice. Interestingly, certain other effects, such as alterations in thymus cellularity, were only found in mice exposed to TCE during gestation. In contrast, expansion of memory/activation cell subset of peripheral CD4^+^ T cells were only found in mice exposed to TCE during early life. Different windows of developmental TCE exposure can have different functional consequences.

## 1. Introduction

The chlorinated hydrocarbon and industrial solvent trichloroethylene (TCE) is a widespread environmental contaminant. As noted in a 2011 IRIS report, the EPA has concluded that “there is substantial potential for environmental exposure to TCE as its improper disposal has resulted in the widespread contamination of groundwater and soil” [1]. Contact with TCE may be elevated for people living near waste facilities, where TCE is released, residents of some urban or industrialized areas, or individuals using TCE-containing products. Regardless of whether TCE exposure is oral, dermal, or inhalation-based, the chemical is rabidly absorbed and distributed. Based on the likelihood of exposure together with likely negative health impact TCE is consistently ranked 16th out of 275 chemicals on the CERCLA list of hazardous chemicals.

One of the predominant human health effects associated with TCE exposure is immunotoxicity, most notably the development of autoimmunity and other types of hypersensitivity diseases. Chronic TCE exposure in adults (both occupational and environmental) has been linked to a variety of autoimmune diseases including systemic lupus erythematosus, scleroderma, hepatitis, and diabetes [2–10]. In addition, there are many cases in recent years of a TCE-induced hypersensitivity disorder that targets the skin and liver [11, 12]. Alterations in CD4^+^ T cells are often found to be an effect biomarker in patients suffering from TCE-induced immunotoxicity [2, 13–15].

Most studies of TCE-induced autoimmunity or hypersensitivity in humans have focused on adult exposure to the higher concentrations of TCE that are most commonly found in the workplace. The type of low-level TCE exposure people may experience through drinking water contamination is generally thought to be risk-free. However, there is evidence that the developing immune system is especially sensitive to even low-level immunotoxicants. A recent review compared early versus adult exposure to several immunosuppressive toxins including lead and tributyltin in animal models [16]. In all cases, sensitivity was greater if exposure occurred during development. In fact, immune suppression in developmentally-exposed offspring often occurred at doses that were ineffective in adults. Developmental sensitivity to toxicants has also been found in humans [17, 18]. This includes evidence that adult onset autoimmune disease can be triggered by pre- and early postnatal toxicant exposure [19, 20].

Developmental exposure to TCE in humans is not uncommon; one study showed that 100% of breast milk samples from 4 US urban areas had detectable levels of TCE [21]. Gestational and early life TCE exposure has primarily been examined for its neurotoxicity [22]. However, children continuously exposed for 3–19 years beginning *in utero* to a water supply contaminated with solvents [with TCE being the predominant toxicant (267 ppb)] had altered ratios of T cell subsets and increased levels of autoantibodies [2].

We have studied a direct cause and effect relationship between TCE exposure and immunotoxicity in a mouse model. Adult exposure to TCE primarily altered effector CD4^+^ T cells, with little effect on CD8^+^ T cells, B cells, or *T*_reg_ cells. The effects of TCE on CD4^+^ T cells were seen after only 4 weeks and included expansion of activated/memory (CD44^hi^ CD62L^lo^) CD4^+^ T cells and altered cytokine production [23–25]. Adult TCE exposure seemed to have minimal effect on thymus cellularity. After chronic adult exposure (26–32 weeks) the TCE-induced alterations in CD4^+^ T cells led to T cell-mediated liver inflammation identical to that seen in idiopathic autoimmune hepatitis (AIH) in humans [24, 26].

We have also examined mice following continuous TCE exposure beginning *in utero* and then encompassing lactation as well as an additional 4 weeks of direct exposure. This continuous exposure to TCE at concentrations lower than that used for adult exposure altered thymocyte cellularity and modified the phenotype and function of peripheral CD4^+^ T cells [27].

Although informative, studies of continuous toxicant exposure do not reveal which windows of exposure have the most impact on the developing immune system. For example, it has been shown that early gestational exposure to certain toxicants such as lead are not as likely to suppress Th1 function as late gestational exposure [28]. This suggests that within the developmental period there exists particular windows of relative susceptibility and resistance [29]. Thus, knowing more about the functional outcomes of particular developmental windows of TCE exposure is needed to accurately estimate risk and to plan interventions. This study was designed to compare central and peripheral immune system alterations in mice exposed to TCE during two crucial windows of immune development and toxicant sensitivity, namely, gestation [GD0 to birth (PND0)] and early life (PND0 to PND49).

## 2. Materials and Methods

### 2.1. Mice and TCE Exposure. 

Developmental exposure to TCE has been described [30]. Basically, breeding pairs of 8-week-old MRL+/+ mice (Jackson Laboratories, Bar Harbor, ME) were established. In one experiment immediately following detection of pregnancy, GD0, the females were divided (following stratified randomization) into 3 groups and given water with 0, 0.01, or 0.1 mg/mL TCE. Controls were given water containing only 1% Alkamuls EL-620, the reagent used to solubilize the TCE. All the drinking water was Ultrapure unchlorinated to assure that chlorination by-products do not confound the results. The TCE-containing drinking water was changed 3 times/week to offset degradation of TCE. Maternal exposure to TCE-containing drinking water ended at birth in this experiment. The female pups that underwent the gestation only exposure to TCE were examined at PND49. As observed previously maternal toxicity was not noted at the level of body weight, mating index, fertility index, sex ratio of litters, gestational length, and food and water consumption.

In the second experiment, the female breeders were not exposed to TCE during pregnancy, but were given TCE-containing drinking water immediately after giving birth. Thus, the pups were exposed to TCE from PND0 to PND21 via lactation. Once the female pups were weaned at PND21 they were exposed to TCE directly in their drinking water for the duration of the experiment. The female pups from this early life exposure were sacrificed at PND49.

Both female breeders and resulting pups in both experiments were weighed weekly and water consumption was monitored. All studies were approved by the Animal Care and Use Committee at the University of Arkansas for Medical Sciences.

When the female pups were sacrificed at PND49 pooled spleen cell suspensions and thymus cell suspensions from paired mice from each litter (*n* = 5–7 litters/treatment group) were examined by flow cytometry. In addition, CD4^+^ T cells were isolated from the pooled spleen cell suspensions and stimulated with immobilized anti-CD3 antibody and anti-CD28 antibody for 24 hours as described [25]. Culture supernatants were then collected for cytokine evaluation, and the activated CD4^+^ T cells were frozen for subsequent qRT-PCR analysis. In addition, adherent macrophages isolated from pooled peritoneal exudates from 2-3 mice/litter were incubated for 20 hours in medium alone or in the presence of LPS (1 *μ*g/mL) and IFN-*γ* (100 units/mL). Approximately 80% of adherent peritoneal exudate cells (PEC), regardless of treatment group, expressed the transmembrane protein F4/80, a marker of mature macrophages (data not shown). Culture supernatants from the peritoneal macrophages were then collected for cytokine evaluation. RLT Lysis Buffer (Qiagen Sciences, Germantown, MD) was then added directly to the remaining adherent cells before freezing for subsequent qRT-PCR analysis.

### 2.2. qRT-PCR. 

Fluorescence-based quantitative reverse transcriptase polymerase chain reaction (qRT-PCR) was conducted using RNA isolated from activated CD4^+^ T cells, peritoneal macrophages or thymocytes. The details for conducting qRT-PCR, including quality control and reference gene selection, have been described previously [31]. The primers used are described in Table 1.

### 2.3. Phenotypic Analysis of Spleen and Thymus Cells

The phenotypic analysis of 30,000 events per sample was carried out using a CyFlow ML (Partec GmbH, Munster, Germany) as described previously using monoclonal antibodies from BD Biosciences or eBioscience [32], and the data were presented as mean percentage ± standard error. Fluorescence Minus One controls and isotype Ig controls were included.

### 2.4. Cytokine Analysis

The culture supernatants from the activated CD4^+^ T cells or peritoneal macrophages were examined using READY-SET-GO ELISA kits for mouse IL-2, IL-6, or TNF-*α* from eBioscience, San Diego, CA.

### 2.5. Statistics

The data are presented as means and standard deviations. Assays were conducted using samples from 5–7 individual litters per treatment group. Comparisons between values obtained from controls and different treatment were made using a Student's *t-*test. The threshold for statistical significance was set at *α* = 0.05.

## 3. Results 

### 3.1. Gross Changes Induced by TCE Exposure

**Figure 1:**
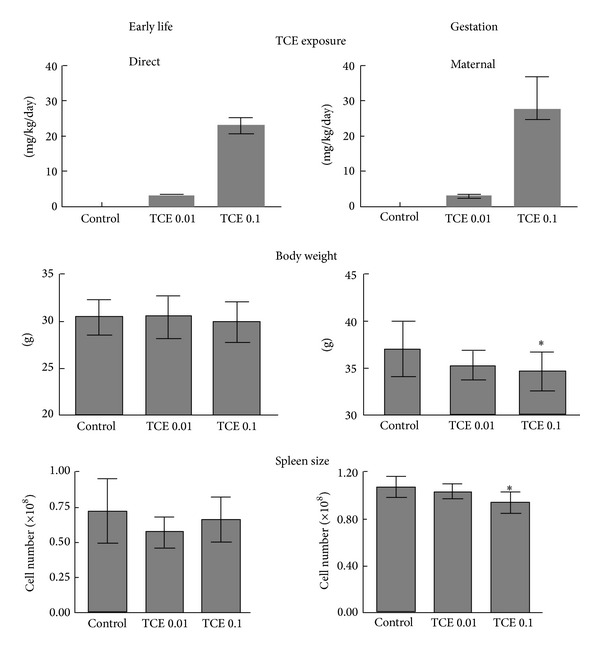
Characteristics of two windows of developmental TCE exposure. Female mice were exposed to TCE (0.01 or 0.1 mg/mL) during gestation only, or during early life only (lactation and 4 weeks of direct exposure). All of the developmentally-exposed mice were examined at PND49. For the gestation only exposure, maternal TCE ingestion based on water consumption was presented. For the early life exposure direct TCE ingestion of the pups postweaning based on water consumption was presented. Body weight and spleen cell numbers (mean ± SD) were determined at PND49. *Significantly different (*α* < 0.05) compared to control values.

Two windows of TCE developmental exposure were examined in female MRL+/+ mice. The mice were either exposed to TCE during gestation (GD0-PND0) or during early life (PND0-PND49). All of the mice were assessed at 7 weeks of age (PND49) for a variety of immune parameters. TCE was added at 0.01 or 0.1 mg/mL to the drinking water of the dams and/or the pups. Based on water consumption, the resulting TCE exposures were described in Figure 1. Many of the alterations in CD4^+^ T cells associated with adult-only direct exposure to TCE were found after 4 weeks [23, 25, 33]. Thus, the PND49 end point can be used to test how the effects of gestational or early life exposure compared to the effects of a direct 4-week adult-only exposure.

Similar to adult exposure to TCE, the early life exposure in the current study did not alter the weight of the female pups at PND49 (Figure 1). In contrast, female pups exposed to the highest concentration of TCE during gestation were significantly smaller than controls. Spleen cell numbers in the same mice were also lower than those of controls. Spleen cell numbers were not altered in mice exposed to TCE during early life only. Thus, TCE exposure during gestation rather than early life, did induce some gross changes in body weight and spleen size.

### 3.2. Developmental TCE Exposure and Changes in Macrophage Function

**Figure 2:**
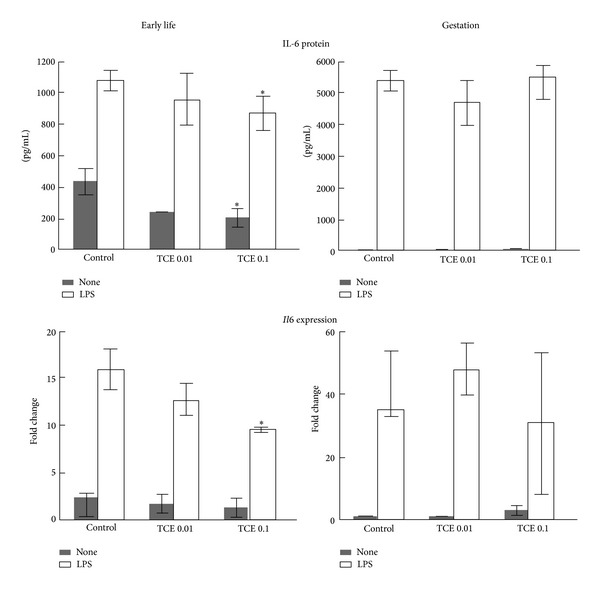
TCE inhibited IL-6 production and *Il6* expression in macrophages. Peritoneal macrophages were incubated with LPS and IFN-*γ* following isolation from untreated control mice or from mice exposed to TCE (0.01 or 0.1 mg/mL) during gestation only or during early life only. Culture supernatants were examined for IL-6 (mean ± SD). *Il6* gene expression was examined in the same peritoneal macrophages. The data represents the mean ± SD. *Significantly different (*α* < 0.05) compared to control values.

Potential TCE-induced alterations in peripheral immune function were examined. These included spleen cellularity and functional activity of key cellular components of chronic inflammation, namely, macrophages and CD4^+^ T cells. IL-6 is a pleiotropic cytokine that has proinflammatory, anti-inflammatory and growth factor properties. Adult exposure to TCE has been shown to suppress macrophage production of IL-6 [34]. In the current study early life exposure to TCE at 0.1 mg/mL similarly inhibited IL-6 production at both the protein and gene expression level (Figure 2). In contrast, gestational exposure to TCE did not suppress IL-6 production by peritoneal macrophages.

### 3.3. Developmental TCE Exposure and Changes in CD4^+^ T Cell Gene Expression

**Figure 3:**
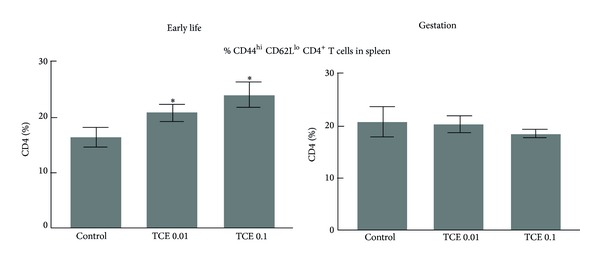
TCE enhanced the percentage of memory/activated CD4^+^ T cells following early life exposure. Spleen cell suspensions from PND49 mice that had been exposed to TCE during gestation only or during early life only were examined by flow cytometry to determine the percentage of CD4^+^ T cells that expressed high levels of CD44 and low levels of CD62L. *Significantly different (*α* < 0.05) compared to control values.

Early-occurring changes in peripheral CD4^+^ T cells have been observed following adult and continuous exposure to TCE. These include expansion of the activation/memory subset of peripheral CD4^+^ T cells and increases in the production of T cell-derived cytokines [24]. These potential alterations were assessed in the current study. TCE did not alter the percentages of total splenic CD4^+^ T cells, CD8^+^ T cells, or B cells, regardless of concentration or window of exposure (data not shown). However, similar to both adult and continuous TCE exposure, early life exposure to TCE at 0.01 or 0.1 mg/mL did increase the percentage of CD44^hi^  CD62L^lo^ CD4^+^ T cells, the population of CD4^+^ T cells considered to represent a memory/activated CD4^+^ T cell phenotype (Figure 3). Gestational exposure did not increase the percentage of activated/memory CD4^+^ T cells in the periphery at PND49.

Splenic CD4^+^ T cells isolated from control and TCE-treated mice were activated *in vitro *prior to an examination of gene expression.Compared to baseline gene expression in unstimulated CD4^+^ T cells, activation *in vitro* increased expression of almost all genes in all CD4^+^ T cells (data not shown). To simplify comparison the gene expression of *in vitro* stimulated CD4^+^ T cells from TCE-treated mice was compared to that of similarly activated CD4^+^ T cells from control mice. This comparison yielded some subtle but significant differences. As observed previously following adult exposure early life exposure to TCE increased expression of *Ifng* and* Il2* but had little effect on *Il4 *(Table 2). Gestational exposure, unlike early life exposure, did not increase the expression of these cytokine genes in activated peripheral CD4^+^ T cells. Thus, once again, early life exposure, unlike gestational exposure, mirrored what has been observed in adult and continuous TCE exposure.

Although CD4^+^ T cells from the two windows of developmental TCE exposure differed in their expression of cytokine genes, they demonstrated remarkably similar expression profiles for *Iap* and *Muerv. *These two genes encode for retrotransposons Intracisternal A particle and murine endogenous retrovirus. The expression of these two retrotransposons is kept in check by epigenetic processes, predominantly DNA methylation [35]. Events that cause global DNA hypomethylation tend to increase expression of *Iap* and *Muerv*, while DNA hypermethylation tends to suppress baseline expression of the retrotransposons. In this study both gestational and early life exposure to the lower concentration of TCE inhibited expression of Iap and MuERV in peripheral CD4^+^ T cells.

DNA methylation is regulated by DNA methyltransferases, a family of enzymes that catalyze the transfer of a methyl group to DNA. This family encompasses *Dnmt1* that is thought to participate in maintenance DNA methylation, and *Dnmt3a*, involved in *de novo* DNA methylation in response to external stimuli [36]. At PND49, neither TCE exposure appreciably altered expression of *Dnmt1* or *Dnmt3a* in CD4^+^ T cells. Thus, the downregulation of *Iap *and *Muerv* in CD4^+^ T cells from mice developmentally exposed to TCE, regardless of gestation or early life, could not be explained by an increase in the expression of *Dnmt1* or *Dnmt3a*.

### 3.4. Developmental TCE Exposure and Changes in CD4^+^ T Cell Function

**Figure 4:**
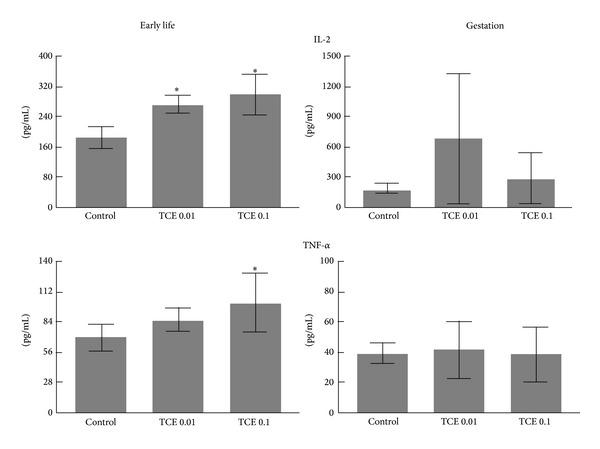
TCE altered cytokines in CD4^+^ T cells from mice exposed to TCE during early life. Cytokine levels in culture supernatants from CD4^+^ T cells prepared as described in Figure 4 were examined. *Significantly different (*α* < 0.05) compared to control values.

In addition to gene expression, cytokine production was examined as a potential marker of TCE-induced alterations in CD4^+^ T cell function. Early life exposure to TCE, similar to adult exposure, increased the production of certain cytokines including IL-2 by CD4^+^ T cells (Figure 4). Gestational exposure alone was not sufficient to increase cytokine production in the peripheral CD4^+^ T cells.

### 3.5. Developmental TCE Exposure and Thymus Cellularity

**Figure 5:**
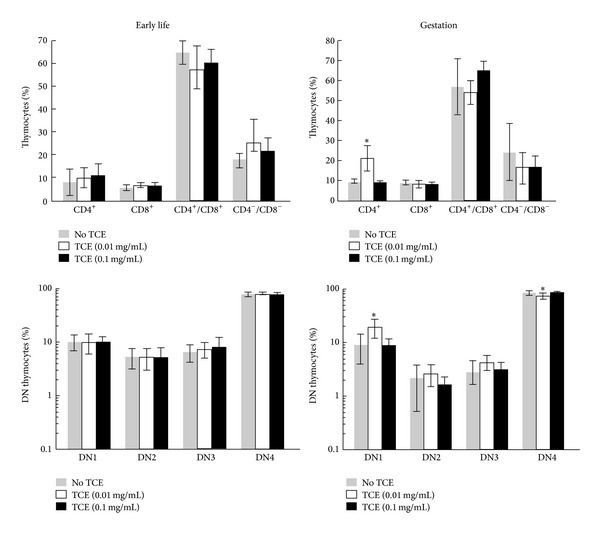
TCE altered thymus cellularity in mice exposed to TCE during gestation. Thymocyte suspensions from PND49 mice that had been exposed to TCE during gestation only or during early life only were incubated for 18 hours and then examined by flow cytometry to determine the percentage of single positive, double negative, and double positive cells. In addition, after gating on the double negative cell population, the cells were stained with anti-CD44 and anti-CD25 Abs and labeled as DN1 (CD44^+^CD25^−^), DN2 (CD44^+^CD25^+^), DN3 (CD44^−^CD25^+^), and DN4 (CD44^−^CD25^−^). *Significantly different (*α* < 0.05) compared to control values.

**Figure 6:**
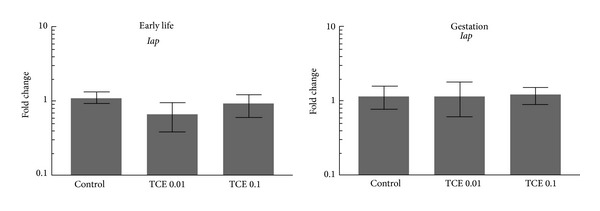
TCE did not inhibit *Iap* in thymocytes from either window of developmental TCE exposure. Gene expression was examined in thymocytes incubated for 18 hours following isolation from untreated control mice or from mice exposed to TCE during gestation only, or during early life only. The data represents the mean ± SD. *Significantly different (*α* < 0.05) compared to control values.

The effects of developmental TCE exposure on central immune function were also examined. The number of total thymocytes was not altered by TCE exposure during gestation or early life (data not shown). However, the composition of thymic subsets was altered in one set of the TCE-exposed mice. Exposure to the lower concentration of TCE during gestation increased the percentage of thymic single positive CD4^+^ T cells (Figure 5). The same mice also demonstrated an increase in the percentage of double negative population 1 (DN1) thymocytes as well as a decrease in percentage of DN4 thymocytes. Unlike gestational exposure early life exposure to TCE did not change the percentages of the different thymocyte subsets measured. Thus, in terms of thymocyte cellularity early life exposure generated results that resembled that of adult exposure, while gestational exposure generated results that most resembled that of previously documented continuous exposure [27]. The thymocytes were then incubated for 18 hours to promote gene expression. Interestingly, neither gestational nor early life exposure to TCE altered *Iap* expression in the thymus, unlike peripheral CD4^+^ T cells (Figure 6). This decreases the likelihood that the altered thymus cellularity of the gestationally exposed mice could be attributed to global changes in DNA methylation.

### 3.6. Developmental TCE Exposure and Thymus Gene Expression

The expression of genes that regulate thymic apoptosis and development were examined in an attempt to explain the altered cellularity in the thymi of mice gestationally exposed to the lower concentration of TCE. In line with the unaltered *Iap* expression in the thymi of mice gestationally exposed to TCE, the expression of enzymes that regulate DNA methylation, namely, *Dnmt1* and *Dnmt3a*, were also unaltered by TCE in the thymus (Table 3). Deficiencies in thymic expression of the gene for the proapoptotic protein Bim or its downstream protein Bax can cause a defect in the negative selection process [37]. Neither of these genes was altered by TCE exposure in the thymus. Survivin, a protein originally described as an inhibitor of apoptosis, has more recently been shown to be required for maturation of DN thymocytes to DP thymocytes [38]. Even though gestational TCE exposure did seem to block an early stage of DN thymocyte differentiation, it did not alter expression of *Survivin*. Gestational exposure to TCE did not alter expression of *Fas*. However, both concentrations of TCE did suppress expression of *Fasl*, the gene that encodes for the ligand for *Fas*, and an important mediator of activation-induced apoptosis. Thus, the increase in SP CD4^+^ thymocytes and DN1 thymocytes may be attributable to a decrease in apoptosis mediated by a TCE-induced decrease in the expression of *Fasl*. However, since the altered cellularity was only found in thymi of mice gestationally exposed to 0.01 mg/mL TCE, while the decrease in *Fasl* was found in mice exposed to either concentration of TCE, the role of *Fasl* in the altered cellularity needs to be clarified.

## 4. Discussion 

The concordance rate for developing an autoimmune disease in identical twins demonstrates the involvement of an ill-defined genetic susceptibility [39]. To mimic this requirement “autoimmune-prone”, MRL+/+ mice were used in our studies. Late in life, MRL+/+ mice can spontaneously develop a relatively mild lupus-like disease, as well as other autoimmune disorders such as Sjogren's syndrome and T cell-infiltrating pancreatitis [40, 41]. Young female MRL+/+ mice, with their propensity for autoimmunity but absence of overt disease, make a good model to test the immunostimulatory capacity of developmental TCE exposure. Others have similarly used “autoimmune-prone” mouse strains to test the developmental immunotoxicity of chemicals such as dioxin, bisphenol A, and mercury [42–44].

The exposure associated with the highest TCE concentration of 0.01 mg/mL (10 ppm) is considerably lower than acceptable human occupational exposure. The EPA's Maximum Contaminant Level for public drinking water is 5 ppb, but TCE has been found at levels up to 1.4 ppm [45] In humans exposed to TCE-contaminated water the amount ingested is only a fraction of the TCE absorbed via inhalation and dermal contact [46]. Since TCE exposure in the mice will be limited to ingestion it could be argued that mouse exposure to 10 ppm is within the range of possible human environmental exposure.

For essentially all of the parameters tested the early life exposure to TCE induced immune system changes similar to adult exposure, albeit at much lower concentrations. On the other hand, the results obtained from gestational only exposure to TCE were similar to those documented after continuous exposure to TCE. Gestational exposure to TCE caused alterations in thymic cellularity and effects on body weight and spleen size. In contrast, gestational TCE exposure had little effect on peripheral CD4^+^ T cells, at least the parameters measured at PND 49. Unlike gestational exposure, early life exposure to TCE had no effect on thymic cellularity but was able to increase the percentage of activated/memory CD4^+^ T cells in association with increased production of cytokines. This implies that exposure to TCE even as early as PND0 had more of an impact on deployed peripheral CD4^+^ T cells than on the thymus. It is possible that lactational exposure to TCE at the concentrations used was not sufficient to impact the still-developing thymus, and that the effects observed following early life exposure were primarily attributable to the direct TCE exposure begun at PND21. An additional study of lactational only exposure to TCE is needed to resolve this question. Immune development in mice correlates well with immune development in humans, albeit with different kinetics [47]. One difference is that the perinatal immune system in mice is even more immature than that of humans. This means that the immune maturation that occurs during human gestation is commensurate with that found in mice at weaning at PND21. Thus, lactation only exposure in mice would correspond with late gestation in humans.

Taken together, it would seem that the individuals most susceptible to the most wide-ranging effects of TCE on the central and peripheral immune system are those exposed to the chemical during gestation as well as early life. Such a scenario in humans could occur when contaminated drinking water for both mother and child results in indirect TCE exposure during gestation and lactation and then direct exposure after weaning. However, it should be noted that, although the two different windows of TCE developmental exposure had distinct effects on peripheral and central T cells, both windows of exposure inhibited expression of retrotransposons in peripheral CD4^+^ T cells. This implies that both gestation and early life are times during which epigenetic changes can be induced. Whether these changes manifest themselves in functional alterations that promote TCE-induced hypersensitivity later in life needs to be determined. Epigenetic processes can regulate several aspects of normal CD4^+^ T cell function including Th1/Th2 differentiation [48], cytokine production [49], and maintenance of Treg cells [50]. There are also several pieces of evidence that epigenetic processes regulate the activity of self-reactive CD4^+^ T cells that mediate autoimmune disease [51–53]. In view of the connection between epigenetic alterations and CD4^+^ T cell autoreactivity, the need to further investigate the capacity of TCE to induce these alterations becomes even more important.

How TCE impacts CD4^+^ T cells is still being defined. Earlier studies showed that the CD4^+^ T cell-altering effects of TCE were most likely mediated by its primary oxidative metabolite trichloroacetaldehyde hydrate (TCAH) [32, 33]. As an aldehyde TCAH can form a functionally active chemical interaction known as a Schiff base with amines on the surface of the CD4^+^ T cells [54]. The possibility that TCAH forms a Schiff base with a costimulator receptor such as CD28, and thus provides bystander costimulation for antigen-activated CD4^+^ T cells is being investigated. How this mechanism would impact thymocytes during development is unclear. In adults, TCE metabolism to TCAH is primarily mediated by cytochrome P450 2E1 (CYP2E1), and to a lesser extent by CYP1A1, and occurs primarily in the liver. However, since CYP1A1, unlike CYP2E1, can be detected in GD7 in the thymus [55], it is possible that CYP1A1 generates TCAH in the thymus of mice developmentally exposed to TCE.

Characterizing TCE-induced developmental immunotoxicity may be part of an important big picture. Immune dysfunction in the form of hypersensitivity disorders are among the most common medical conditions affecting children in the USA. Many of these appear to be increasing. For example, more children are being diagnosed with type I diabetes [56], childhood Graves' disease [57], and lupus nephritis [58] than ever before. Similarly, the CDC reports that the prevalence of food allergies increased from 3.4% in 1997–1999 to 5.1% in 2009–2011 [59]. The prevalence of skin allergies also increased from 7.4% in 1997–1999 to 12.5% 2009–2011. It is possible that the increase in childhood hypersensitivity is linked to increased exposure to environmental chemicals with potential developmental toxicity. More than 85,000 synthetic chemicals have been developed in the past 75 years, and only about 20% of the 3,000 most widely used chemicals have ever been tested for general developmental toxicity let alone developmental immunotoxicity [60]. As part of NHANES 2003-2004, it was found that after adjusting for covariates levels of many chemicals in pregnant women were increased compared to nonpregnant women [61]. Certain polychlorinated biphenyls, organochlorine pesticides, polycyclic aromatic hydrocarbons and many other chemicals were detected in 99-100% of pregnant women. Some of these chemicals cross the placenta and mediate developmental immunotoxicity. Developmental immunotoxicity is based on the premise that during the maturation of the immune system toxicant exposure results in a qualitative or quantitative difference in the effect or a greater persistence of effect. This increased sensitivity may be attributed to (i) a greater chemical exposure per pound of body weight, (ii) immature metabolic systems unable efficiently clear toxicants, (iii) developing immune system that is easily disrupted, and (iv) more time to develop chemical-induced immune-mediated diseases. Studying developmental immunotoxicity of chemicals such as TCE with proinflammatory effects may provide important clues to the etiology of idiopathic autoimmune disease.

## 5. Conclusion

Early life exposure to TCE induced changes in peripheral CD4^+^ T cell commensurate with those found in mice exposed to TCE as an adult. Gestational TCE exposure induced changes in central immune function similar to that observed following continuous exposure. However, the two windows of exposure did have one effect in common, namely, the downregulation of retrotransposon expression in CD4^+^ T cells. Since the effects induced by the two developmental windows of TCE exposure were associated with TCE concentrations lower than those effective in adult, exposure the differential responses are functionally important.

## Figures and Tables

**Table 1:** Primer sequences used for qRT-PCR.

Gene		Primer sequences 5′ to 3′	Tm (°C)
*Aire *	Sense	AGATCGCGGTGGCCATAG	57.4
	Antisense	TCGTGGTCGGCTAGAGCAT	58.4
*Bax *	Sense	TTGCTGATGGCAACTTCAACTGGG	60.2
	Antisense	TGTCCAGCCCATGATGGTTCTGAT	60.4
*Bim *	Sense	CTGTGTAATGTGCCCTACTGTTTC	55.8
	Antisense	GGAAGAGAACCAGCCACTACC	57.3
*CD70 *	Sense	TGCTGGTGGTGTTTATTACTGTG	55.4
	Antisense	CTCTGGTCCGTGTGTGAAGG	57.7
*Cdkn1a *	Sense	AATCCTGGTGATGTCCGACCTGTT	60.2
	Antisense	GTGACGAAGTCAAAGTTCCACCGT	59.4
*Dnmt1 *	Sense	TGATAAGGAGGACAAGGAGAATGC	56.4
	Antisense	CACCGCCAAGTTAGGACACC	58.3
*Dnmt3a *	Sense	CAGCACCATTCCTGGTCATGCAAA	60.2
	Antisense	TCCTGTGTGGTAGGCACCTGAAAT	60.2
*Fas *	Sense	CGCCCGCTGTTTTCCC	57.6
	Antisense	GCAAGCACCAGAGGCAGG	59.4
*FasL *	Sense	GGCTGGGTGCCATGCA	59.4
	Antisense	GGCACTGCTGTCTACCCAGAA	59.2
*IAP *	Sense	GCACCCTCAAAGCCTATCTTAT	54.6
	Antisense	TCCCTTGGTCAGTCTGGATTT	55.8
*Ifng *	Sense	AGCTCATCCGAGTGGTCCAC	59.1
	Antisense	AGCAGCGACTCCTTTTCCG	57.8
*Il2 *	Sense	CCCAAGCAGGCCACAGAATTGAAA	60.2
	Antisense	AGTCAAATCCAGAACATGCCGCAG	59.9
*Il4 *	Sense	AGCCATATCCACGGATGCGACAAA	60.8
	Antisense	AATATGCGAAGCACCTTGGAAGCC	60.0
*Il6 *	Sense	AGAGGAGACTTCACAGAGGATACC	57.1
	Antisense	CATTTCCACGATTTCCCAGAGAAC	56.1
*Muerv *	Sense	TGGTGGTCGAGATGGAGGTTA	57.5
	Antisense	CCGTGAATGGTGGTTTTAGCA	55.8

**Table 2:** Gene expression in CD4^+^ T cells from mice exposed to TCE during gestation or early life.

	Early life			Gestation		
	Control	TCE 0.01	TCE0.1	Control	TCE 0.01	TCE 0.1
*Iap *	1.0 ± 0.17	0.12 ± 0.02	0.42 ± 0.36	1.00 ± 0.1	0.43 ± 0.05	0.72 ± 0.19
*Muerv *	0.95 ± 0.26	0.6 ± 0.11	0.76 ± 0.23	1.0 ± 0.15	0.44 ± 0.04	0.95 ± 0.26
*Il2 *	1.0 ± 0.1	1.49 ± 0.2	1.31 ± 0.1	0.91 ± 0.1	0.97 ± 0.29	0.66 ± 0.1
*Il4 *	1.0 ± 0.17	1.1 ± 0.35	1.4 ± 0.34	1.1 ± 0.43	1.47 ± 0.75	1.44 ± 0.93
*Cdkn1a *	1.0 ± 0.03	0.9 ± 0.07	0.92 ± 0.07	1.05 ± 0.38	1.62 ± 0.64	1.41 ± 1.1
*Ifng *	0.91 ± 0.16	0.95 ± 0.11	1.31 + 0.22	1.32 ± 1.0	1.0 ± 0.81	1.42 ± 1.11
*Dnmt1 *	1.0 ± 0.24	1.05 ± 0.12	0.89 ± 0.22	1.11 ± 0.51	1.58 ± 0.36	1.73 ± 0.71
*Dnmt3a *	0.98 ± 0.02	1.08 ± 0.07	1.09 ± 0.07	0.98 ± 0.51	1.12 ± 0.35	1.2 ± 0.87

Gene expression was examined in splenic CD4^+^ T cells incubated with anti-CD3 and anti-CD28 Abs for 24 hours following isolation from untreated control mice or from mice exposed to TCE during gestation only, or during early life only. The data represents the mean ± SD. *Significantly different (*α* < 0.05) compared to control values.

The bolded text represents values that are significantly different from control values (*α* < 0.05).

**Table 3:** Gene expression in thymi of mice exposed to TCE during gestation.

	Gestation		
	Control	TCE 0.01	TCE 0.1
*Bax *	1.04 ± 0.14	0.78 ± 0.24	1.06 ± 0.75
*Bim *	1.02 ± 0.15	0.82 ± 0.3	0.87 ± 0.21
*Dnmt1 *	1.0 ± 0.3	1.16 ± 0.5	1.03 ± 0.25
*Dnmt3a *	1.14 ± 0.56	1.17 ± 0.46	0.93 ± 0.26
*Survivin *	1.3 ± 0.97	0.53 ± 0.47	0.68 ± 0.42
*Aire *	1.1 ± 0.58	0.71 ± 0.09	0.76 ± 0.51
*Fas *	0.96 ± 0.49	0.83 ± 0.46	0.78 ± 0.28
*Fasl *	1.06 ± 0.42	0.4 ± 0.25	0.45 ± 0.28

Gene expression was examined in thymocytes incubated for 18 hours following isolation from untreated control mice or from mice exposed to TCE during gestation only.

The data represents the mean ± SD. *Significantly different (*α* < 0.05) compared to control values.

The bolded text represents values that are significantly different from control values (*α* < 0.05).
